# Transcriptome Analysis Reveals Candidate Genes Related to Anthocyanin Biosynthesis in Different Carrot Genotypes and Tissues

**DOI:** 10.3390/plants9030344

**Published:** 2020-03-09

**Authors:** Geng Meng, Sabine K. Clausen, Søren K. Rasmussen

**Affiliations:** Department of Plant and Environmental Sciences, University of Copenhagen, Thorvaldsensvej 40, 1871 Frederiksberg C, Denmark; gengm@plen.ku.dk (G.M.); kbg184@alumni.ku.dk (S.K.C.)

**Keywords:** *Daucus carota* L., apiaceae, transcriptome, gene expression, anthocyanin, MYB, bHLH, glutathione S-transferase, acyltransferase

## Abstract

Black carrots are characterized by a significant amount of anthocyanins, which are not only a good source of natural food colorant, but can also provide many health benefits to humans. In the present work, taproots of different carrot genotypes were used to identify the candidate genes related to anthocyanin synthesis, with particular a focus on R2R3MYB, bHLH transcription factors, and glutathione S-transferase gene (*GST*). The RNA-sequencing analysis (RNA-Seq) showed that *DcMYB6* and *DcMYB7* had a genotypic dependent expression and they are likely involved in the regulation of anthocyanin biosynthesis. They were specifically upregulated in solid black taproots, including both black phloem and xylem. *DcbHLH3* (LOC108204485) was upregulated in all black samples compared with the orange ones. We also found that *GST1* (LOC108205254) might be an important anthocyanin transporter, and its upregulated expression resulted in the increasing of vacuolar anthocyanin accumulation in black samples. Moreover, high performance liquid chromatographic (HPLC) analysis and liquid chromatography coupled to mass spectrometry (LC-MS) were used to identify the individual anthocyanin in the purple tissues of two carrot cultivars. The results showed that five main anthocyanin compounds and the most abundant anthocyanin were the same in different tissues, while the second-highest anthocyanin between three tissues was different, even in the same cultivar. In conclusion, this study combined anthocyanin profiles and comparative transcriptomic analysis to identify candidate genes involved in anthocyanin biosynthesis in carrots, thus providing a better foundation for improving anthocyanin accumulation in carrots as a source of colorants.

## 1. Introduction

Anthocyanins, one of the phenolic compounds, are derived from the flavonoids biosynthesis pathway. Anthocyanins are ubiquitous in plants, a wide variety of colors result from the anthocyanin synthesis. Apart from their colorant properties, anthocyanins also play an important role in preventing UV radiation, responding to cold and drought stress, promoting pollination, and defending against bacterial and fungal pathogens [1,2]. 

Wild carrots (*Daucus carota*) are indigenous to Europe, Northern Africa, and parts of western Asia [3] and have whitish or pale yellow roots, while the original carrots domesticated in Persia and Arabia with purple and yellow roots [4,5]. The domesticated carrots are divided into Western and Eastern groups [6], black or purple carrots (*Daucus carota* L. *ssp. sativus var. atrorubens* Alef.) the latter are characterized by a relatively high amount of anthocyanins. In recent decades, black carrots have attracted more and more attention, as they are not only a good source of natural food colorant [7,8], but also comprise a variety of antioxidant components that provide health benefits to humans [9,10]. There are many different purple carrots cultivars, which can be differentiated by their xylem color, such as ‘Antonina’ with white/yellow xylem, ‘Purple Haze’ and ‘Beta Sweet’ with orange xylem [11]. In purple carrots, the anthocyanin composition has been well studied and reported by several researchers, cyanidin-3-xylosyl (feruloylglucosyl)-galactoside is the major one [12,13,14]. The acylated anthocyanins were shown to account for more than half of the total anthocyanin content in many black carrot cultivars [14]. In addition, purple carrots are considered as desirable material for food colorant production, since these acylated cyanidin derivatives are more stable than nonacylated ones with the increasing of storage time [15,16]. 

Anthocyanin biosynthesis is one of the branches of the flavonoid biosynthesis pathway. Most of the structural genes involved in this pathway have been intensively studied in many plant species [17]. Firstly, phenylalanine ammonia-lyase (PAL) transforms phenylalanine to trans-cinnamic acid. Cinnamate 4-hydroxylase (C4H) and 4-coumarate-CoA ligase (4CL) sequentially convert trans-cinnamic acid into 4-coumaroyl-CoA. After that, chalcone synthases (CHS), chalcone isomerase (CHI) and flavonoid 3 hydroxylase (F3H) participate in the early steps of this pathway. The downstream genes in the pathway were dihydroflavonol 4-reductase (*DFR*), leucoanthocyanidin dioxygenase (*LDOX*) and UDP-glucose: flavonoid 3-glucosyltransferase (*UFGT*) [1]. Previous reports showed that these genes (except for *UFGT*) participated in anthocyanin biosynthesis in the taproots of black carrots [18,19]. Acyltransferases, such as SCPL (Serine Carboxypeptidase-Like) in carrots, catalyze the formation of acylated anthocyanins [20,21]. Moreover, anthocyanins were transported and accumulated into vacuoles by transporters [1], including the glutathione S-transferase (GST) family, the ATP-binding cassette (ABC) and multidrug and toxic compound extrusion (MATE) families [20,21,22]. The first GST in relation to anthocyanin was reported in maize [23], anthocyanin accumulation was reduced due to the loss-of-function of *Bronze2* (*Bz2*). Subsequently, homologs of the *Bz2* gene, including *Arabidopsis TT19* [24], petunia *AN9* [22], and grape *VvGST1* and *VvGST4* [25], were found to transport anthocyanins into vacuoles. Nevertheless, to date, no direct evidence has been found for the role played by GSTs in conjugating anthocyanin in carrot.

Expression of anthocyanin biosynthesis pathway genes is controlled by the MYB-bHLH-WD40 ‘MBW’ complex, MYB transcription factors (TFs) are the major contributors to the regulation of this pathway [26]. In maize, the *C1* gene regulated the anthocyanin contents through the interaction with structural genes [27]. Subsequently, *MdMYB10* and *MdMYB1* in apples [28,29], *VvMYBA1* and *VvMYBA2* in grapevines [30], and *PyMYB10* in pears [31] were found to be the transcriptional activators of anthocyanin biosynthesis. bHLH proteins of the MBW complex also play an important role in the anthocyanin biosynthesis in many plants. In lotus, *NnMYB5*, interacting with *NnbHLH1*, acted as a transcription activator of anthocyanin synthesis [32]. In apples, *MdbHLH3* was identified as an activator of anthocyanin accumulation in fruit [33]. In strawberries, *FabHLH3* was responsible for the modification of proanthocyanin contents [34]. *GtbHLH1* in gentian flowers interacted with *GtMYB3* to improve the promoter activity of structural genes in tobacco [35]. In potatoes, *StbHLH1* has been reported to be involved in anthocyanin biosynthesis [36].

Despite a comprehensive study of anthocyanin-related MYB, bHLH TFs and *GST* on other species, there is still limited information about these genes in carrot taproots. According to previous reports, five MYB transcription factors, named *DcMYB1* to *DcMYB5*, have been shown to be involved in the transcriptional regulation of *DcPAL1* to *DcPAL4* in carrot cell suspension cultures. Among them, *DcMYB1* could stimulate the expression of *DcPAL1* to defend against UV-B irradiation [37], and *DcMYB3* and *DcMYB5* play important roles in the 2,4-D regulated expression of *DcPAL3* in anthocyanin accumulation [38]. It is worth noting, however, that in vitro protoplasts were used as the resource of materials in the above studies. It may not be applicable when it comes to in planta conditions. The expression pattern of *DcMYB6* and *DcMYB7* were recently reported to be positively correlated with anthocyanin accumulation in carrot taproots [39,40]. It should also be noted that the black carrot genetic background and developmental stages used in that study were completely different from the materials used here. More important, in our study, the mechanism of coloration was also studied with a particular focus on the xylem and phloem of different carrot cultivars. There are various differences of expressed genes under different genetic backgrounds and growth stage, which will provide a new perspective on the study of carrot pigmentation in terms of the tissue, genotype, and stage specificity. 

In this study, three carrot cultivars with different pigmentation patterns in the phloem and xylem of their taproots were selected in order to investigate the anthocyanin biosynthesis mechanism in a tissue-specific manner. HPLC analysis combined with LC-MS was used to identify and relatively quantify individual anthocyanins in different black tissues. Although the anthocyanin profile has been well studied by several researchers, there are no reports so far in terms of tissue-specific study. A comprehensive analysis integrating RNA-Seq, gene expression analyses, and orthologous and phylogenetic analysis strongly suggested that *DcMYB6* and *DcMYB7*, one *bHLH* gene, and one *GST* gene were involved in anthocyanin pigmentation in carrot taproots in this study. 

## 2. Results 

### 2.1. Total Anthocyanin Accumulation during Four Growth Stages

**Figure 1:**
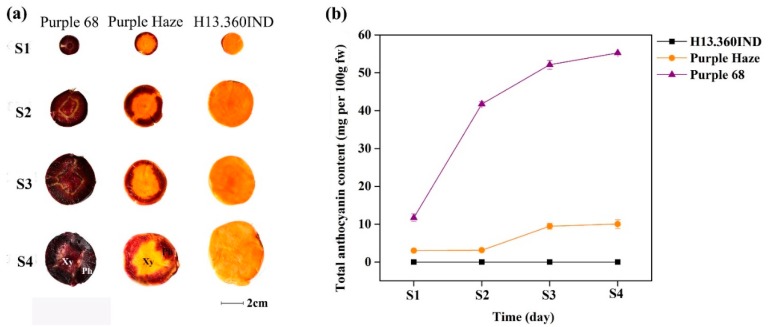
Differential pigment accumulation analysis in three carrot cultivars (‘Purple 68′, ‘Purple Haze’ and ‘H13.360IND’). (**a**) Four growth stages of three carrot cultivars. S1, S2, S3, and S4 represent 4 growth stages of harvested carrots at 60, 80, 100 and 120 days after sowing, respectively. Xy and Ph represent xylem and phloem, respectively. (**b**) Total anthocyanin content in four growth stages. The data were expressed as mg of cyanin equivalents per g fresh weight.

To understand the changes that take place in the anthocyanin content of carrots during development and ripening, we categorized developing carrot taproots into four growth stages: S1, S2, S3, and S4, representing 60, 80, 100 and 120 days after sowing, respectively (Figure 1a). The total anthocyanin content (TAC) was determined (Figure 1b) using the pH differential method [41]. As shown in Figure 1a, three cultivars displayed distinct coloration, the purple-pigmented area of phloem in ‘Purple 68′ and ‘Purple Haze’ grew from S1 to S4. The purple color of the xylem in ‘Purple 68′ increased, while the xylem of ‘Purple Haze’ stayed orange across all the stages.

‘Purple 68′ showed the highest TAC followed by ‘Purple Haze’ across all the stages. No anthocyanin was detected in the orange carrot cultivar ‘H13.360IND’. The TAC of ‘Purple 68′ increased sharply from stage S1 to S2 and then reached a plateau at S3 and S4. TAC of ‘Purple Haze’ had slightly increasing from S1 to S4 (Figure 1b). The TAC of ‘Purple 68′ and ‘Purple Haze’ in S4 were highest compared to the other stages, and there was no significant increase from S3 to S4. The carrot taproots from S4 (120 days after sowing) were chosen for RNA isolation and sequencing.

### 2.2. Characterization of Anthocyanins in the Carrot Phloem and Xylem 

In our study, the purple samples are the phloem of ‘Purple 68′ and ‘Purple Haze’, and the xylem of ‘Purple 68′ at S4. LC-MS was carried out to identify and relatively quantify individual anthocyanins of purple samples based on retention time, mass, and spectra, and comparisons with reported data from black carrot [8,42]. By calculating the percentage peak area from HPLC measurements, a typical anthocyanin profile of a black carrot root extract was identified in Supplementary File S1. The five major compounds were cyanidin 3-xylosylgalactoside, cyanidin 3-xylosyl (glucosyl) galactoside, cyanidin 3-xylosyl (feruloylglucosyl/coumaroylglucosyl/sinapoylglucosyl) (Supplementary File S1). The feruloyl derivative of cyanidin xylosylglucosylgalactoside was the predominant pigment in all the samples, cyanidin 3-xylosylgalactoside was the second-highest anthocyanin in the phloem of ‘Purple 68′, while cyanidin 3-xylosyl (sinapoylglucosyl) galactoside was the second-highest in the xylem of ‘Purple 68′ and phloem of ‘Purple Haze’. 

### 2.3. An Overview of Transcriptome Sequencing Datasets

To further identify the molecular mechanism of anthocyanin biosynthesis in carrots, RNA-Seq was used to investigate changes in comprehensive gene expression in carrot taproots at S4 displaying different pigmentation patterns. Approximately 41.74–46.30 million paired-end clean reads were generated (Table 1). The clean Q30 base rates, a key parameter that represents the quality of sequenced bases, were around 93–94% in all the libraries, suggesting high-quality sequencing. Of the total clean reads, 60.06–66.61% were uniquely mapped, that is to say they were mapped to only one position in the reference transcriptome (Table 1). These results indicated that the RNA-Seq data obtained could be used for this study. 

### 2.4. Identification of Differentially Expressed Genes between the Phloem and Xylem of ‘Purple 68′ and ‘Purple Haze’ 

**Figure 2:**
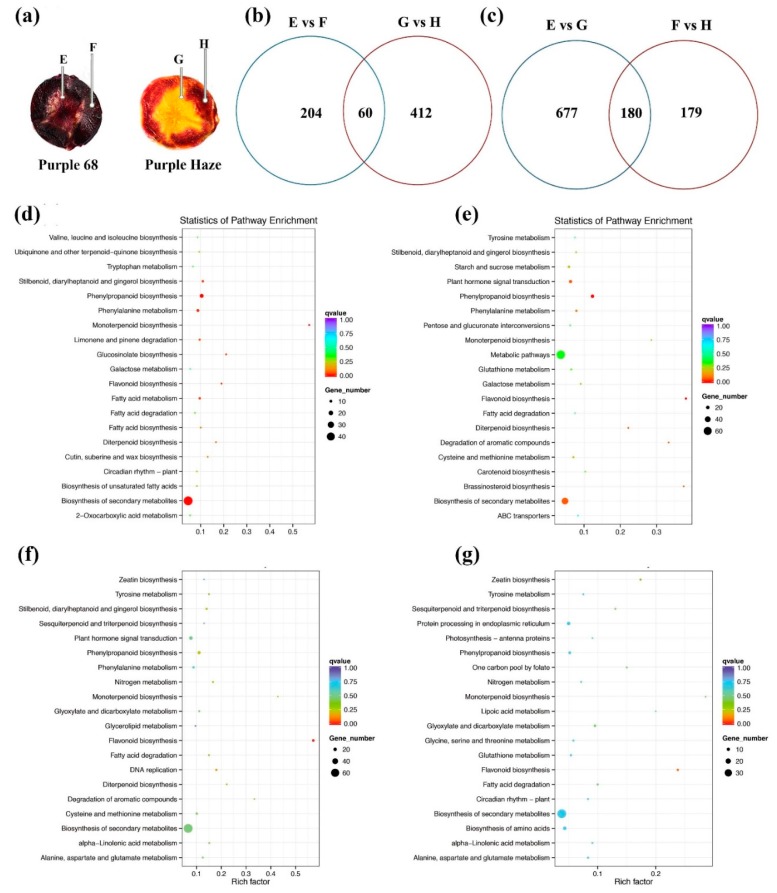
KEGG pathway analysis of different of carrots tissues (**a**) Phloem and xylem tissue in ‘Purple 68′ and ‘Purple Haze’. (**b**) Venn diagram of DEGs in the comparison of E vs. F and G vs. H. (**c**) Venn diagram of DEGs in the comparison of E vs. G and F vs. H. (**d**–**g**) Scatterplot of the top 20 enriched KEGG pathways of E vs. F, G vs. H, E vs. G and F vs. H, respectively. Rich factor is the ratio of DEGs counts to this pathway in the annotated genes counts. q ≤ 0.05 indicates significant enrichment. E (xylem of ‘Purple 68′), F (phloem of ‘Purple 68′), G (xylem of ‘Purple Haze’), and H (phloem of ‘Purple Haze’).

In the present study, the differentially expressed genes (DEGs) between the phloem and xylem of ‘Purple 68′ and ‘Purple Haze’ were investigated to find genes that were expressed through a tissue-specific way. KEGG pathway analysis of these DEGs were performed, which provides a foundation for underlying pigmentation mechanism through systematically analyzing the biological processes (Supplementary File S2). Firstly, the aim was to understand the mechanism that made ‘Purple 68′ and ‘Purple Haze’ display differential coloration between phloem and xylem. There were 264 genes identified as DEGs when comparing the xylem to the phloem (E vs. F) in ‘Purple 68′, and 472 DEGs in ‘Purple Haze’, respectively (Figure 2a). Of these DEGs, 60 genes were shared by both comparisons (Figure 2a). There were 61 and 70 pathways detected from the phloem and xylem comparisons in ‘Purple 68′ and ‘Purple Haze’, respectively (Supplementary File S2). The top twenty significantly enriched pathways are shown in the KEGG scatter plot (Figure 2). In the comparison of xylem and phloem in ‘Purple 68′, the top three enriched pathways were ‘monoterpenoid biosynthesis, Rich Factor = 0.57′, ‘glucosinolate biosynthesis, Rich Factor = 0.21′ and ‘flavonoid biosynthesis, Rich Factor = 0.19′ (Figure 2c). In ‘Purple Haze’, the most significantly enriched pathway was ‘flavonoid biosynthesis, Rich Factor = 0.38′, followed by ‘brassinosteroid biosynthesis, Rich Factor = 0.37′ and ‘degradation of aromatic compounds, Rich Factor = 0.33′ (Figure 2d). Therefore, various KEGG pathways were found, the flavonoid biosynthesis pathway was significantly enriched in both comparisons, while other plant secondary metabolite pathways were differentially involved in regulating the pigmentation between two cultivars. 

Secondly, to investigate the candidate genes that made the deep purple and purple phloem, and also the purple and orange xylem in ‘Purple 68′ and ‘Purple Haze’, respectively, 857 DEGs were found by comparing the xylem (E vs. G), and 205 DEGs when comparing the phloem (F vs. H) between two cultivars. Among these DEGs, 180 genes were commonly expressed in both comparisons (Figure 2b). In the comparison of xylems (E vs. G), a total of 101 pathways were enriched (Supplementary File S2), of which the top five enriched pathways were ‘flavonoid biosynthesis’, ‘monoterpenoid biosynthesis’, ‘degradation of aromatic compounds’, ‘diterpenoid biosynthesis’ and ‘lipoic acid metabolism’ (Figure 2e). In the comparison of phloems (F vs. H), 83 pathways were enriched, the top five being ‘monoterpenoid biosynthesis’, ‘flavonoid biosynthesis’, ‘lipoic acid metabolism’, ‘zeatin biosynthesis’ and ‘indole alkaloid biosynthesis’ (Figure 2f). KEGG analysis revealed that the main activated genes resulting in different purple colored phloem or different colored xylem (purple and orange) in ‘Purple 68′ and ‘Purple Haze’, which might be involved in tissue-specific coloration, were predominately involved in the biosynthesis of secondary metabolite pathways, hormone signal transduction and other cellular components and molecular function-related terms.

### 2.5. Differentially Expressed Structural Genes in Anthocyanin Biosynthesis Pathway

**Figure 3:**
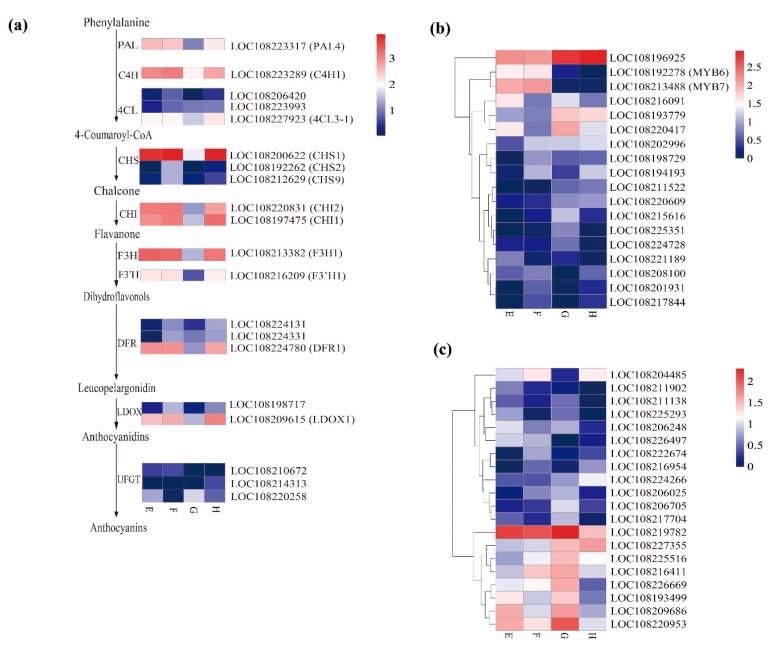
Expression pattern of differently expressed genes (DEGs) involved in anthocyanin synthesis. (**a**) Expression pattern of structural genes involved in anthocyanin synthesis. (**b**) Expression pattern of MYB involved in the different tissues. (**c**) Expression pattern of bHLH involved in the different tissues. The value of log10 (FPKM + 1) is represented by the depth of color, with red representing upregulation and blue representing downregulation. PAL, phenylalanine ammonia-lyase; C4H, cinnamate 4-hydroxylase; 4CL, 4-coumarate-CoA ligase; CHS, chalcone synthase; CHI, chalcone isomerase; F3H, flavonoid 3 hydroxylase; F3′H, flavonol 3′ hydroxylase; DFR, dihydroflavonol 4-reductase; LAR, leucoanthocyanidin reductase; UFGT, UDP glucose: flavonoid-O-glucosyltransferase. E (xylem of ‘Purple 68′), F (phloem of ‘Purple 68′), G (xylem of ‘Purple Haze’) and H (phloem of ‘Purple Haze’).

According to the analysis above, the different coloration in these cultivars was regulated by the biosynthesis of secondary metabolites, especially the flavonoid. Anthocyanin, belonging to the class of flavonoid compounds, is responsible for the formation of the purple color-specific carrot. In the present study, 20 putative structural genes were identified. Of these, 10 genes were highly upregulated in all the purple color-specific tissues; the others had very low expression levels among all the samples (Figure 3a; Supplementary File S3). These 10 genes were *PAL4*, *C4H1*, *4CL3-1*, *CHS1*, *CHI1*, *CHI2*, *F3H1*, *F3′H1*, *DFR1* and *LDOX1* (Figure 3a). Therefore, the expression levels of most structural genes related to flavonoid metabolic pathways were higher in purple tissues than in orange ones, leading to higher anthocyanin accumulation. Based on the reported data, of the three *CHS* genes, LOC108200622 was annotated as *DcCHS1* (GenBank ID: AJ006779.1), and LOC108192262 and LOC108212629 corresponded to *DcCHS2* (GenBank ID: D16255.1) and *DcCHS9* (GenBank ID: D16256.1), respectively [43,44]. It is noticeable that *DcCHS1* was the most significantly expressed gene in all purple tissues. In addition, one *SCPL* gene, named *DcSCPL1* [20], which catalyze anthocyanin acylation in carrot, was highly expressed in all the purple tissues (Supplementary File S3).

### 2.6. Candidate R2R3MYB and bHLH TFs Involved in Anthocyanin Biosynthesis

Regulation of the anthocyanin pathway is achieved through the interaction of MYB, bHLH and WD40-repeat proteins [45,46]. WD40s and bHLHs stimulate a large number of genes responding to changing environmental conditions, while MYBs determine the specific genes to be regulated, which means MYBs are indeed the main regulator of anthocyanin biosynthesis in plants. Here, the focus was on the individual MYB and bHLH TFs that are involved in anthocyanin biosynthesis.

Eighteen *MYB* genes were identified as DEGs during the tissue-specific comparisons (Figure 3c; Supplementary File S3). Specifically, two *MYB113-like* genes (namely LOC108192278 and LOC108213488), also known as *DcMYB6* (DCAR_000385) and *DcMYB7* (DCAR_010745), were highly and solely expressed in ‘Purple 68′, while their expressions were almost undetected in ‘Purple Haze’ (Figure 3b, Supplementary File S3). The results indicated that both of these genes were related to anthocyanin accumulation in a genotype-specific manner. In addition, the expression level of LOC108208100 was higher in purple samples (E, F, and H), than in orange section of ‘Purple Haze’. On the contrary, the expressions of two *MYB* genes, including LOC108196925 and LOC108193779 were much higher in ‘Purple Haze’ than in ‘Purple 68′. Both of them were predicted as MYB1R1-like transcription factors and may negatively regulate anthocyanin content in these two carrot genotypes.

In the present study, 20 *bHLHs* were identified as DEGs across all the comparisons (Figure 3c; Supplementary File S3). The transcripts of one *bHLH* gene, LOC108204485, were detected at high levels in all the purple tissues but at much lower levels in the orange samples (Figure 3c). In addition, three *bHLH* genes, including LOC108193499, LOC108209686 and LOC108220953 were highly expressed in the xylem of ‘Purple Haze’ and ‘Purple 68′ as compared with phloem in ‘Purple Haze’. They might be related to anthocyanin accumulation in a tissue-specific manner.

### 2.7. GST Involved in Anthocyanin Transport in Carrots

**Figure 4:**
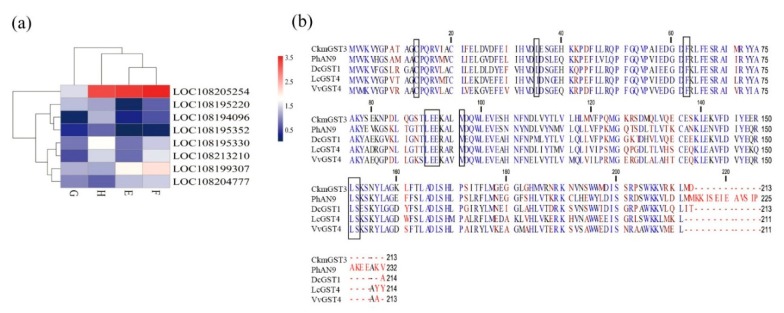
Expression patterns of *DcGST*s and deduced amino acid sequences analysis. (**a**) Heatmap of DEGs of GST across four comparisons in different tissues. Expression values of four libraries are presented as normalized log10 (RPKM + 1). Red and blue colors indicate upregulated and downregulated transcripts, respectively. E (xylem of ‘Purple 68′), F (phloem of ‘Purple 68′), G (xylem of ‘Purple Haze’) and H (phloem of ‘Purple Haze’). (**b**) Multiple alignments of deduced amino acid sequences of DcGST1 (LOC108205254) with other functionally anthocyanin-related GSTs. Black boxes represent amino acid residues of GST involved in anthocyanin transporting [47]. The GenBank accession numbers are *PhAN9* (CAA68993), *CkmGST3* (BAM14584), *VvGST4* (AAX81329), and *LcGST4* (ALY05893).

The GST transporters (GSTs) which are responsible for transporting anthocyanins into the vacuole have been studied previously. In our study, eight GST genes were identified as DEGs across all the comparisons (Figure 4a; Supplementary File S3). Among them, one *GST* gene (LOC108205254) was significantly upregulated in all the purple tissues as compared with the orange one (Figure 4a; Supplementary File S3). Here, this *GST* gene was named *DcGST1*. Multiple alignments with other functional GSTs showed that this *DcGST* protein contained the conserved residues confirmed for anthocyanin-related GSTs (Figure 4b). 

### 2.8. Validation of RNA-Seq Results with Quantitative Real-Time PCR (qRT-PCR) 

**Figure 5:**
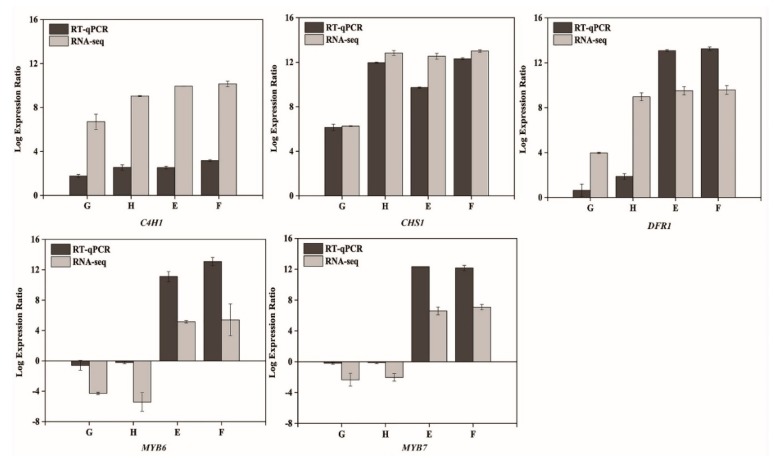
Validation of the RNA-seq results by qRT-PCR. Bars show means of Log2 FPKM data and Log2 qRT-PCR data of two and three biological replicates, respectively. FPKM means the expected number of fragments per kilobase of transcript sequence per Millions base pairs sequenced. E (xylem of ‘Purple 68′), F (phloem of ‘Purple 68′), G (xylem of ‘Purple Haze’) and H (phloem of ‘Purple Haze’).

RNA samples isolated for the RNA sequencing were also used to perform the qRT-PCR analysis to validate the results of RNA-Seq. Five DEGs were randomly selected for the analysis of qRT-PCR. FPKM value was calculated for all samples, and the Log2 ratio of FPKM was used to reflect the fold changes compared with CT. These results are shown in Figure 5, these genes in RNA-seq and qRT-PCR shared the similar expression patterns. Taken together, RNA-Seq results were confirmed by the qRT-PCR analysis.

## 3. Discussion

The genetic background of plants is a crucial factor in determining anthocyanin biosynthesis and here the three carrot genotypes used in our study were characterized with distinct colors in their phloems and xylems. HPLC analysis was performed in order to determine the individual anthocyanin of purple samples, namely the xylem and phloem tissue of ‘Purple 68′ and the phloem tissue of ‘Purple Haze’. Five main anthocyanins were found to match those reported in other studies [11,13,48]. Cyanidin 3-xylosyl (feruloylglucosyl) galactoside was identified as the most abundant anthocyanin of carrots, while the second-highest anthocyanin between the three tissues was different. Therefore, our results are the first demonstration that the anthocyanin profiles were not the same in different tissues of the same cultivar taproot.

A series of structural genes control the anthocyanin biosynthesis directly, most of them have been studied in carrot [18,19,37,43,44,49]. In the present study, 10 genes including *PAL4*, *C4H1*, *4CL3-1*, *CHS1*, *CHI1*, *CHI2*, *F3H1*, *F3′H1*, *DFR1* and *LDOX1* may be involved in the anthocyanin biosynthesis in purple carrots, as these genes showed higher expression levels in purple tissues than in orange tissues. In agreement with our results, by comparing the gene expression levels of 13 structural genes in purple carrots and one non-purple carrot [18], we identified nine of them—including *CHS1*, *CHI1*, *F3H1*, *F3′H1*, *DFR1*, *LDOX1/LDOX2*, *PAL3*, *CA4H1*, and *4CL1*—as being associated with purple root coloration, and all of them as coinciding with the genes found by RNAseq in our study. They did not, however, analyze *CHI2* (also upregulated in the present study). It was reported that four of the five genes (*CHS1*, *F3H1*, *DFR1*, *LDOX*, and *PAL3*) [19] coincided with the purple root-upregulated genes found in the present work, *PAL3* being the exception. Furthermore, in a more recent study, it was reported that six genes (*CHS1*, *CHI1*, *F3H1*, *F3′H1*, *DFR1*, *LDOX1* and some *UDGP* genes) were found to be upregulated in purple carrot roots [50]. Six of them coincide with our study, except for *UDGP* genes. Three *UFGT-like* genes were not differentially expressed in any of the comparisons, which is in agreement with a previous report [19], the expression level of *UFGT* was always relatively constant, regardless of the color and developmental stage of the carrots.

In our study, *PAL4* was upregulated in all the purple tissues, while in two studies [18,19], but not in [50], *PAL3* was the upregulated one. Altogether, this data suggests that *PAL* and *UFGT* genes seem to be upregulated in specific purple-rooted genetic backgrounds.

In Arabidopsis, the deficiency of CHS protein in mutants resulted in a decrease in the anthocyanin contents in the cotyledons and hypocotyls, while the seedlings of wild-type were rich in anthocyanins [51]. In our study, only *DcCHS1* was highly expressed in purple tissues compared with orange tissues, while *DcCHS2* and *DcCHS9* had very low expression in all the samples. This indicated that *DcCHS1*, but not *DcCHS2* and *DcCHS9*, was involved in anthocyanin biosynthesis in carrots. This is consistent with the data presented in [18,43], showing that *DcCHS1* expression was highly correlated with the formation of anthocyanin. Therefore, *DcCHS1* is believed to play a crucial role in carrot pigmentation. Purple carrots accumulate large quantities of acylated anthocyanins in their roots, however, there are no carrot genes annotated as anthocyanin acyltransferases previously. Recently, it was found that *DcSAT1* could be activate by *DcMYB7* to regulate the glycosylation and acylation of anthocyanins [52]. In our study, one *SCPL* gene (LOC108214129) expression was much higher in all the purple samples than in the orange one. Very recently, it was reported that *DcSCPL1* (namely LOC108214129), was always expressed in association with anthocyanin pigmentation in the root, which is in agreement with our results [53]. They found that *DcSAT1* and *DcSCPL1* protein sequences are identical, therefore from here we use *DcSCPL1* instead of *DcSAT1* and LOC108214129. The expression level of *DcSCPL1* was higher in the phloem than in the xylem of ‘Purple 68′, which may explain the difference of anthocyanin profiles in different tissues of the same cultivar.

R2R3MYB TFs were reported as major regulators that promote anthocyanin biosynthesis [28,54,55]. Recently, a cluster of *MYB* genes were in two regions on chromosome 3, namely P1 and P3, controlling pigmentation in carrots [50]. To be specific, eight *MYB* genes (*MYB6*, *MYB7*, *MYB8*, *MYB9*, *MYB10*, *MYB11*, *MYB14*, and *MYB15*), and two *MYB* genes (*MYB12* and *MYB13*) were located in the P3 and P1 regions, respectively. In agreement with results in [40], it was found that *MYB11* co-localized with the mapped trait for petiole pigmentation making this gene responsible for anthocyanin pigmentation only in petiole [50]. Other genes except for *MYB6* and *MYB7*, were not differently expressed in our study. In the present study, *DcMYB7* and *DcMYB6* had the same expression pattern, which were highly upregulated in ‘Purple 68′, while almost undetected in ‘Purple Haze’ (Figure 3b, Supplementary File S3). *DcMYB7* had higher expression than *DcMYB*6, and it may play a more important role in anthocyanin accumulation. It was reported [52] that the loss of purple pigmentation in carrot taproots resulted from the knocking out of *DcMYB7*. In another study [40], it was reported that *DcMYB7* was an important gene for anthocyanin synthesis, independent of the genotype of carrot in their study, which is the opposite of our results. We found that *DcMYB7* was a genotype-dependent expressed gene and was only expressed in solid purple genotypes (in both purple phloem and xylem). The present results support the findings [56] that *DcMYB7* was differentially expressed in solid purple cultivar.

*DcMYB6* was reported [39] to regulated anthocyanin synthesis in the carrot taproot. Increased anthocyanin contents were found due to the overexpression of this gene in Arabidopsis. However, later it was reported [40] that *DcMYB6* was not the essential gene regulating anthocyanin accumulation in carrots used in their study because *DcMYB6* is downregulated in the purple roots of one carrot cultivar. In our study, *DcMYB6* was highly expressed in the purple phloem and xylem of ‘Purple 68′. Its expression was positively correlated to the anthocyanin accumulation, which is consist with the results in [39]. Another study show that *DcMYB6* may regulate root anthocyanin pigmentation in specific genetic backgrounds [50]. It is noteworthy that the purple carrot cultivars used in our study and in [39] were purple in the phloem and xylem, while the carrot cultivars used in the later study [40] had purple phloem, but orange xylem. These facts, taken together, indicate that it is possible that *DcMYB6* and *DcMYB*7 are involved in genotype-dependent regulation of anthocyanin biosynthesis in carrots. They were specifically upregulated in solid purple carrots, i.e., both phloem and xylem were purple.

In addition, it was reported that the *MYB3-like* gene (LOC108208100) continued to be upregulated [56] in all purple callus or tissue samples, except for solid purple cultivar ‘Superblack’ (purple phloem and xylem). However, LOC108208100 was upregulated in the solid purple cultivar ‘Purple 68′ in our study, it may therefore be related to anthocyanin accumulation in a purple color-specific way.

In our study, we found one *bHLH* gene, namely LOC108204485, was upregulated in all purple samples, but almost undetected in the orange sample. It was reported [52] that the *bHLH* gene (LOC108204485), named as *DcbHLH3* (QEA09235.1) in their study, interacted with *DcMYB7* using yeast two-hybrid assays. Therefore, we hypothesize that the *bHLH* gene (LOC108204485) might regulate anthocyanin accumulation through the interaction between MYB TFs in purple samples.

*GST1* (LOC108205254) was highly expressed in all the purple-pigmented tissues compared with the orange tissue. It shares 69.81% amino acid sequence identity with GST for anthocyanin accumulation in *Cyclamen persicum×Cyclamen purpurascens* (Genebank id: BAM14584.1) [47]. In addition, it shares 72.64% amino acid sequence identity with GST in *Actinidia chinensis var*. chinensis (Genebank id: PSS21435.1) [57]. Moreover, it possesses the typical anthocyanin-related amino acid residues found in dicotyledons [47]. Therefore, the *GST1* may play a positive role in transporting anthocyanin into vacuoles in purple tissues to increasing the anthocyanin concentration. To the authors’ knowledge, the GST gene family has not been studied in relation to carrot anthocyanin biosynthesis.

## 4. Materials and Methods

### 4.1. Plant Materials

Three different types of cultivars were used in this study: ‘H13.360IND’, ‘Purple 68′, and ‘Purple Haze’. The seeds were sown and grown in greenhouses at the University of Copenhagen, Frederiksberg, Denmark. Characteristics of the cultivars: ‘Purple Haze’ is purple colored with an orange core, ‘Purple 68′ is purple colored, and ‘H13.360IND’ is yellow colored. The seeds of black carrot cultivars ‘Purple Haze’ and ‘Purple 68′ were purchased from Bejo Zaden (bj) in The Netherlands, and orange cultivar ‘H13.360IND’ was provided by Nunhems in The Netherlands. Taproots were harvested at 60, 80, 100, and 120 days after sowing, and then sliced into 0.2 cm discs along the horizontal axis, approximately 1 cm from the taproot’s top. Ten taproots per cultivar were mixed respectively, with three biological replicates used for each sample in HPLC and LC-MS analysis, and two biological replicates in RNA-Seq. All the materials were frozen in liquid nitrogen, powdered individually and then stored at −80 °C for further study.

### 4.2. Determination of Total Anthocyanin Content and Individual Anthocyanin

The total anthocyanin content (TAC) was determined using the pH-differential method [41]. Briefly, about 0.1 g frozen powder of samples were extracted overnight with 8 mL of 80% methanol at 4 °C. After centrifugation at 10,000× *g* for 20 min, transfer 1 mL extracted solution was transferred to a new tube and the volume was adjusted to 10 mL with potassium chloride buffer, pH 1.0; another 1 mL extracted solution was transferred into a new tube and the volume adjusted to 10 mL with sodium acetate buffer, pH 4.5. For each pH, absorbance was measured using a spectrophotometer Evolution™ 220 (Thermo Scientific, Waltham, MA, USA) at 510 and 700 nm, respectively. The following formula was applied to determine the anthocyanin contents:

C = A × V × n × MW × 100/(ε × m)(1)

where A is the absorbance of the diluted sample, A = (A_510_ − A_700_ nm) _pH 1.0_ − (A_510_ − A_700_ nm) _pH 4.5_,

C is anthocyanin content (mg 100 g^−1^ FW), V is extraction solution volume, n is dilution factor, MW is the molecular weight of cyanidin-3-glucoside: 449.2, Ɛ for molar absorptivity: 30,200, m is the weight of fresh sample used.

In terms of HPLC analysis, the carrot taproot sample (500 mg) was mixed with 70% methanol containing 2% formic acid at 4 °C. A 0.45 μm syringe filter was used for filtering the supernatant. Then the filtered supernatant was used for the HPLC analysis. Phenolic compounds were analysed using HP1200 (Agilent Technology, Palo Alto, CA, USA). The details of the analysis were described in [58]. To complement the HPLC analyses, LC-MS analysis was performed following the method described in [59]. Chromatography was performed on a Dionex UltiMate^®^ 3000 Quaternary Rapid Separation UHPLC focused system (Thermo Fisher Scientific, Germering, Germany).

### 4.3. RNA Extraction and cDNA Synthesis

Total RNA was harvested from the frozen carrot samples with TRIzol reagent (Sigma-Aldrich, Inc., St. Louis, MO, USA) following the manufacturer’s protocol. The RNA quality was identified by a Spectrophotometer NanoDrop 2000C, Thermo Scientific, Waltham, MA, USA), and 4 µL of RNA was loaded on 1% agarose gels to check the degradation and contamination. First-strand cDNA was obtained using M-MuLV Reverse Transcriptase (NEB, Ipswich, MA, USA), according to the manufacturer’s protocol, and stored at −20 °C for qRT-PCR assays.

### 4.4. Library Construction and Transcriptome Sequencing

For the RNA-Seq analysis, 120-day-old carrot taproots of three cultivars were chosen. For ‘H31.360IND’, the whole slice was used; for ‘Purple 68′ and ‘Purple Haze’ the carrot discs were dissected into the xylem and phloem tissue samples. Following this, five RNA-Seq libraries were constructed and labeled as follows: CT (whole slice of ‘H31.360IND’), E (xylem of ‘Purple 68′), F (phloem of ‘Purple 68′), G (xylem of ‘Purple Haze’) and H (phloem of ‘Purple Haze’). Two biological replicates were used for each sample. Therefore, ten libraries were obtained in this study in all. The sequencing of libraries was conducted by Novagene Corporation using Illumina^®^ (NEB, Ipswich, MA, USA) according to the manufacturer’s instruction. Subsequently, the mRNA was purified, and first-strand cDNA was synthesized with M-MuLV reverse transcriptase. Then the following steps were converting the overhangs into blunt ends, preparing for hybridization, and purifying the cDNA fragments. Before PCR, adaptor-ligated cDNA libraries were treated with USER Enzyme (NEB, Ipswich, MA, USA). Then PCR was performed with Phusion High-Fidelity DNA polymerase. Lastly, PCR products were purified, and library quality was assessed on the Agilent Bioanalyzer 2100 system (Agilent Technologies, Santa Clara, CA, USA). The data have been deposited in NCBI’s Gene Expression Omnibus; GEO Series accession number GSE146419 and may be accessed at https://www.ncbi.nlm.nih.gov/geo/query/acc.cgi?acc=GSE146419.

### 4.5. RNA-Sequencing Data Analysis

Clean reads were obtained after the filtering of raw data. Reference genome and gene annotation files of carrot were downloaded from NCBI carrot genome [60]. The index of the reference genome was built using Bowtie v2.2.3. Then paired-end clean reads were aligned to the reference genome using TopHat v2.0.12. The level of gene expression was determined according to the number of fragments per kilobase of exon per million fragments mapped (FPKM). Differential expression analysis of two samples (two biological replicates per sample) was performed using the DESeq R package (1.18.0). The resulting *p*-values were adjusted using the Benjamini–Hochberg approach to control the false discovery rate. Genes with an adjusted *p*-value < 0.05 and log2 (fold change) of 1 by DESeq were assigned as differentially expressed. Additionally, GO enrichment analysis of differentially expressed genes was implemented by the ClusterProfile R package. KOBAS software was used to test the statistical enrichment of DEGs in KEGG pathways.

### 4.6. RT-qPCR Validation

To verify the reliability of the RNA-Seq results, the total RNA of sequenced samples was used to perform qRT-PCR. Five DEGs were chosen and their expression quantified by qRT-PCR on the LightCycler machine using the 5×FIREPol EvaGreen qPCR mix according to the manufacturer’s instructions. The DcActin gene was used as a reference. Primer 3.0 software program was used to design the primer pairs, and the primer list is shown in Supplementary File S4. The relative gene expression levels were determined using the 2^−∆∆CT^ approach. Each RNA sample has three biological repetitions. Each of them was quantified with three technical repeats.

## 5. Conclusions

We have identified candidate genes that are involved in the anthocyanin biosynthesis pathways in purple carrots. RNA-Seq analysis was used to find the DEGs contrasting the colored phloem and xylem of taproot of various cultivars. We propose that some *MYB*, *bHLH*, and *GST* genes are related to anthocyanin biosynthesis in a color or genotype-specific manner. In addition, the anthocyanin profiles in purple carrot taproots were identified from the tissue-specific aspect for the first time. The key impact of this work is that it provides insights into candidate genes that may regulate the anthocyanin biosynthesis in a genotype-specific way. A better understanding of the process of anthocyanin production in carrots should provide new opportunities for genetic improvement and management in the future.

## Figures and Tables

**Table 1:** Summary of RNA-Seq data and reads mapping.

Sample Name	CT	E	F	G	H
Raw reads	45,469,730	47,158,771	47,299,178	42,815,288	45,142,919
Clean reads	44,276,147	46,187,029	46,303,908	41,744,653	43,245,500
Clean bases	6.64	6.93	6.945	6.26	6.49
Q20 (%)	97.595	97.545	97.575	97.615	97.7
Q30 (%)	93.625	93.48	93.56	93.64	93.675
GC content	43.9%	44.855%	44.86%	44.69%	44.715%
Total mapped	28,571,418 (64.37%)	29,128,798 (63.06%)	28,684,092 (61.99%)	28,468,352.5 (68.19%)	28,480,330 (66.06%)
Uniquely mapped	27,830,779.5 (62.70%)	28,343,267 (61.36%)	27,809,975.5 (60.09%)	27,807,121.5 (66.61%)	27,840,397.5 (64.58%)

The different samples for RNA isolation represent CT (whole slice of ‘H13.360IND’), E (xylem of ‘Purple 68′), F (phloem of ‘Purple 68′), G (xylem of ‘Purple Haze’) and H (phloem of ‘Purple Haze’). Q20 (%) and Q30 (%) are the percentages of reads with quality scores of >20 and >30, respectively.

## Supplementary Materials

The following are available online at https://www.mdpi.com/2223-7747/9/3/344/s1, Supplementary file S1. Chromatographic properties of anthocyanins detected in black carrot tissues by HPLC and LC-MS analysis, RT represent retention time. Supplementary file S2. KEGG pathway of comparison between E vs. F, G vs. H, E vs. G and F vs. H. E and F represents xylem and phloem of ‘Purple 68′, respectively. G and H represent xylem and phloem of ‘Purple Haze’, respectively. Supplementary file S3. FPKM value of differentially expressed structural genes, MYB, bHLH TFs and GST genes in different tissues. CT represents orange cultivar ‘H13.360IND’, E and F represents xylem and phloem of ‘Purple 68′, respectively. G and H represents xylem and phloem of ‘Purple Haze’. Supplementary file S4. Primer list for the qRT-PCR.
